# Treatment strategies of hospitalized patients with coronavirus disease-19

**DOI:** 10.18632/aging.103370

**Published:** 2020-06-17

**Authors:** Yaxiong Huang, Chunlin Cai, Jinglei Zang, Jun Xie, Dan Xu, Fang Zheng, Tao Zhan, Kang Huang, Yikai Wang, Xiao Wang, Zhe-Yu Hu, Yapeng Deng, Yuanlin Xie

**Affiliations:** 1 The First Hospital of Changsha City, Changsha 410005, Hunan, China; 2 Changsha Health Vocational College, Changsha 410100, Hunan, China; 3 Emory University Rollins School of Public Heath, Atlanta, GA 30322, USA; 4 ICF, 3 Corporate Square NE., Atlanta, GA 30329, USA; 5 Hunan Cancer Hospital, The Affiliated Cancer Hospital of Xiangya School of Medicine, Central South University, Changsha 410013, Hunan, China; 6 The Forth Hospital of Changsha City, Changsha 410013, Hunan, China

**Keywords:** coronasvirus disease-19, COVID-19, epidemiological and clinical characteristics, treatment strategies, prognosis, outside Wuhan

## Abstract

With the outbreak of coronavirus disease-19 (COVID-19), Changsha faced an increasing burden of treating the Wuhan migrants and their infected patients. This study is a retrospective, single-center case series of the 238 consecutive hospitalized patients with confirmed COVID-19 at the First Hospital of Changsha city, China, from 01/21 to 02/14, 2020; the final date of follow-up was 02/27, 2020. Of 238 patients 43.7% visited Wuhan, 58.4% got in touch with Wuhan people, and 47.5% had contacted with diagnosed patients. 37.8% patients had family members infected. 190 cases had mild / general disease, and 48 cases had severe / critical disease. Compared to mild or general patients, more severe or critical patients visited Wuhan (59.6% vs 40.2%; *P*=0.02) and contacted with Wuhan people (74.5% vs 55.0%; *P*=0.02). All patients received antiviral treatment, including Lopinavir / Ritonavir (29.3%), Interferon (14.6%) and their combination (40.6%), Arbidol (6.7%), Xuebijing (7.1%) and Chloroquine phosphate (1.3%). Severe and critical patients received glucocorticoid, Gamma-globulin and oxygen inhalation. Some received mechanic ventilation support. As of 02/27, 161 patients discharged. The median length of hospital stay was 13 days. The 10-, 14-, 20- and 28-day discharge rate was 19.1%, 42.8%, 65.0% and 76.4%, respectively. No hospital-related transmission was observed.

## INTRODUCTION

Beginning in December 2019, cases of pneumonia with new coronavirus infection began to appear in Wuhan City, Hubei Province, China. Since then, the number of infected cases has increased exponentially. World Health Organization (WHO) has officially named the new coronavirus that caused the outbreak of pneumonia in Wuhan as "coronavirus disease-19 (COVID-19)" [1–3].

On December 8, 2019, the first patient was diagnosed in Wuhan Central Hospital with a history of exposure to the South China Seafood Market [4]. In the following Spring Festival travel rush, COVID-19 spread rapidly in Hubei province, China and even the rest of the world [5]. Five million people left Wuhan before the festival, 65 percent of whom returned to home in Hubei Province and 35 percent scattered throughout the country.

As the nearest metropolis to Wuhan, Changsha city (335 km in distance) has faced great pressure after the outbreak of COVID-19 in Wuhan. Hunan Province is the second largest destination in China for the 5 million people who left from Wuhan. It is estimated that approximately 0.3 million Wuhan people migrate to Changsha before the festival. Fortunately, Changsha has abundant medical resources, including three affiliated Xiangya Hospitals of Central South University, three affiliated hospitals of Hunan University of Traditional Chinese Medicines, five provincial hospitals and five city hospitals. As of February 14, 2020, a total of 238 COVID-19 cases have been confirmed in Changsha, and all of them were isolated and treated in the north hospital of the First Hospital of Changsha City. Of these, 66 cases have met the discharge criterion after intensive treatment as of February 14, 2020. The number of admitted cases ranks 7^th^ and the number of discharged cases ranks 8^th^ in the country.

As for the death rate, Wuhan has the highest death rate to date at 3.52%. Besides the death rates in Hong Kong, Heilongjiang province, Hainan province and Tianjin City at 2.98%, 2.52%, 2.38% and 2.29%, the death rate of most provinces is less than 2%. Therefore, we believe that the imported COVID-19 is a curable and controllable disease with effective treatment. The efficacy of clinical treatment is remarkable. In this study, we summarized the treatment strategy and outcome of the 238 COVID-19 cases admitted in Changsha.

## RESULTS

### Patients’ characteristics at admission

This study included 238 consecutive hospitalized patients with confirmed COVID-19. The median age was 45 years (IQR: 34-59; range: 1-84). 117 (48.7%) were males. 110 (46.2%) were office workers, 33 (13.9%) were retired people, 12 (5.0%) were students or a teacher (only 1), 3 (1.3%) were medical workers (not infected at work site), 16 (6.8%) were freelance workers/ self-employed / sales, 6 (2.5%) were farmer / cooker / train attendant, and the other 57 (24.1%) had no job designation. 104 (43.7%) had a recent history of visiting Wuhan, and their median duration from leaving Wuhan to diagnosis was 10 days (IQR: 7-14). 139 (58.4%) had a history of getting in touch with Wuhan people. 113 (47.5%) had a history of getting in touch with diagnosed patients. 90 (37.8%) had infected family members.

Among these patients, 190 (79.8%) were the mild or general type and were admitted to isolation wards. 48 (20.2%) patients were of severe or critical type and were transferred to the ICU. Among these 48 patients, 21 (43.8%) were mild or general at admission, but then progressed to severe pneumonia and transferred to the ICU within a median of 4 days (IQR, 2-5 days). The median durations from the onset of symptoms to diagnosis and hospital admission were 4 days (IQR: 2-7) and 5 days (IQR: 3-8), respectively (Table 1). Among the 238 patients, 93 (39.1%) had one or more coexisting comorbidities. The most common comorbidities were hypertension (36 [15.1%]), diabetes (15 [6.3%]), chronic hepatobiliary disease (15 [6.3%]), heart disease (HD) (including 8 coronary HD) (11 [4.6%]), and pulmonary disease (including 1 COPD) (10 [4.2%]). The most common symptoms at onset of illness were fever (159 [66.8%]), cough (137 [57.6%]), fatigue (65 [27.3%]), expectoration (49 [20.6%]). Less common symptoms were pharyngalgia, anorexia, chest tightness / pain, chills, dyspnea, myalgia and diarrhea (from 15.5% to 8.4%) (Table 1).

**Table 1:** Patient characters.

	**Total (n=238)**	**Clinical Classification**		*P value
		**Mild / General (n=190)**	**Severe / Critical (n=48)**	
Age, median (IQR), yr	45 (±17), 45 (34, 59)	43 (±17), 41 (31-54)	54 (±15), 54 (44, 66)	<.001
Sex, No (%),				
Female	122 (51.3)	101 (53.2)	21 (43.8)	0.24
Male	117 (48.7)	89 (46.8)	27 (56.3)
Occupations, No (%),				
Office workers	110 (46.2)	89 (47.1)	21 (43.8)	0.70
Retired	33 (13.9)	20 (10.6)	13 (27.1)	0.003
Student (1 teacher)	12 (5.0)	12 (6.3)	0 (0)	0.06
Medical Worker	3 (1.3)	3 (1.6)	0 (0)	0.50
Freelance Worker/ Self-employed /Sales	16 (6.8)	13 (6.9)	3 (6.3)	>.99
Farmer / Cooker / Train Attendant	6 (2.5)	4 (2.1)	2 (4.2)	0.35
None	57 (24.1)	48 (25.3)	9 (18.8)	0.34
Wuhan visit, No (%),	104 (43.7)	76 (40.2)	28 (59.6)	0.02
Left Wuhan to diagnosis, median (IQR), d	10 (7, 14)	10 (7, 15)	8 (7, 12)	0.29
Wuhan people touch, No (%),	139 (58.4)	104 (55.0)	35 (74.5)	0.02
Patient touch, No (%),	113 (47.5)	98 (51.6)	15 (31.9)	0.02
Family members infected, No (%),	90 (37.8)	77 (40.5)	13 (27.1)	0.09
Comorbidities, No (%),				
Hypertension	36 (15.1)	21 (11.1)	14 (29.2)	0.002
Chronic hepatobiliary disease	15 (6.3)	11 (5.8)	4 (8.3)	0.51
Diabetes	15 (6.3)	10 (5.3)	5 (10.4)	0.19
Heart disease (HD) (8 Coronary HD)	11 (4.6)	4 (2.1)	7 (14.6)	0.002
Pulmonary diseases (1 COPD)	10 (4.2)	8 (4.2)	2 (4.2)	>.99
Cerebrovascular disease	8 (3.4)	5 (2.6)	3 (6.3)	0.20
Bone disease	8 (3.4)	6 (3.2)	2 (4.2)	0.66
Gastric disease	7 (2.9)	6 (3.2)	1 (2.1)	>.99
Gynecological (breast) disease (1 pregnancy)	7 (2.9)	5 (2.6)	2 (4.2)	0.63
Chronic kidney disease	4 (1.7)	1 (0.5)	3 (6.3)	0.03
Endocrine	3 (1.3)	1 (0.5)	2 (4.2)	0.10
Blood disease	2 (0.8)	1 (0.5)	1 (2.1)	0.36
Malignancy	2 (0.8)	2 (1.1)	0 (0)	>.99
Sign and symptoms, No (%),				
Fever	159 (66.8)	114 (60.0)	45 (93.8)	<.0001
Cough	137 (57.6)	106 (55.8)	31 (64.6)	0.27
Fatigue	65 (27.3)	44 (23.2)	21 (43.8)	0.004
Expectoration	49 (20.6)	36 (19.0)	13 (27.1)	0.21
Pharyngalgia	37 (15.5)	33 (17.4)	3 (6.3)	0.07
Anorexia	28 (11.8)	20 (10.5)	8 (16.7)	0.24
Chest tightness / pain	23 (9.7)	18 (9.5)	5 (10.4)	0.84
Chilly	22 (9.2)	14 (7.4)	8 (16.7)	0.05
Dyspnea	20 (8.4)	9 (4.7)	11 (22.9)	<.0001
Myalgia	20 (8.4)	13 (6.8)	7 (14.6)	0.08
Diarrhea	20 (8.4)	13 (6.8)	7 (14.6)	0.08
Headache	11 (4.6)	6 (3.2)	5 (10.4)	0.03
Dizziness	9 (3.8)	7 (3.7)	2 (4.2)	>.99
Vomiting	8 (3.4)	7 (3.7)	1 (2.1)	>.99
Nasal discharge	6 (2.5)	6 (3.2)	0 (0)	0.60
Nasal obstruction	6 (2.5)	5 (2.6)	1 (2.1)	>.99
Onset of symptom to, median (IQR), d				
Diagnosis	4 (2, 7)	4 (2, 7)	4 (3, 8)	0.15
Hospital admission	5 (3, 8)	5 (3, 8)	5 (3, 9)	0.22
Arterial pressure difference, median (IQR), mm Hg	48 (43-54)	47 (43, 54)	49 (44, 54)	0.40

Abbreviations: COPD, chronic obstructive pulmonary disease; IQR, interquartile range; HD, heart disease.

* *P* values indicate differences between mild / general type and severe / critical patients. *P* < .05 was considered statistically significant.

Compared with mild or general patients, the severe or critical patients were significantly older (median age, 54 years [IQR, 44-66] vs 41 years [IQR, 31-54]; *P*<.001) and more likely to have coexisting comorbidities, such as hypertension (14 [29.2%] vs 21 [11.1%]; *P*=0.002), heart disease (HD) (7 [14.6%] vs 4 [2.1%]; *P*=0.002), and chronic kidney disease (3 [6.3%] vs 1 [0.5%]; *P*=0.03). Compared to mild or general patients, the severe or critical patients were more likely to report fever, fatigue, chills, dyspnea, and headache. In addition, compared to mild or general patients, more severe or critical patients tended to have visited Wuhan (28 [59.6%] vs 76 [40.2%]; *P*=0.02) and to have gotten in touch with Wuhan people (35 [74.5%] vs 104 [55.0%]; *P*=0.02), but tended to be less likely to be infected by patients (15 [31.9%] vs 98 [51.6%]; *P*=0.02) and family members (13 [27.1%] vs 77 [40.5%]; *P*=0.09). These findings suggested the primary Wuhan infection might be more serious than the secondary infection from patients or family members.

### Temperature, image and laboratory indexes

At admission, more severe or critical patients tended to have high fever. The arterial pressure difference (APD) and vital signs (heart rate and respiratory rate (RR)) did not differ between mild / general patients and severe / critical patients. 16 mild patients had no obvious abnormal chest CT image. 144 (60.5%) patients showed bilateral involvement in a chest scan. In laboratory findings, severe / critical patients had significantly lower lymphocyte count and higher platelet count (Table 2). All severe patients had PaO_2_ / FiO_2_ < 300 mmHg or the oxygen saturation < 93% or the appearance of respiratory distress RR > 30 /min. All critical patients had respiration failure (invasive mechanical ventilation support) or shock or with failure of other organs.

**Table 2:** Temperature, image and laboratory findings of NCIP patients at admission.

	**Normal range**	**Total (n=238)**	**Clinical classification**		*P value
			**Mild / General (n=190)**	**Severe / Critical (n=48)**	
Temperature					
Low fever (37.3°C -38.0°C)	36.3°C–37.2°C	42 (17.6)	24 (12.6)	18 (37.5)	<.001
Medium fever (38.1°C -39.0°C)		12 (5.0)	8 (4.2)	4 (8.3)	0.27
High fever (above 39.0°C)		3 (1.3)	0 (0)	3 (6.3)	0.01
CT image					
Normal		6 (2.5)	6 (3.2)	0 (0)	0.60
Single lung involvement		88 (37.0)	73 (38.4)	15 (31.3)	0.36
Bilateral involvement		144 (60.5)	111 (58.4)	33 (68.7)	0.19
Laboratory findings					
White blood cell count, ×10^9^/L	3.5-9.5	4.6 (3.5, 5.7)	4.7 (3.6, 5.7)	4.5 (2.9, 5.6)	0.06
Neutrophil count, ×10^9^/L	1.8-6.3	2.9 (2.1, 3.7)	2.9 (2.1, 3.6)	3.0 (2.0, 3.8)	0.44
Lymphocyte count, ×10^9^/L	1.1-3.2	1.1 (0.8, 1.6)	1.2 (0.9, 1.7)	0.7 (0.5, 1.4)	<.001
Eosinophil count, ×10^9^/L	0.05-0.50	0.01 (0, 0.05)	0.02 (0.01, 0.06)	0 (0, 0.01)	0.10
Platelet count, ×10^9^/L	125-350	139 (111, 172)	178.5 (145.5, 235)	150 (130, 189)	0.03
Hemoglobin, g/L	110-160	130 (120, 141)	129 (120, 141)	130 (119, 143)	0.99

Abbreviations: CT, computed tomography.

* *P* values indicate differences between mild / general type and severe / critical patients. *P* < .05 was considered statistically significant.

### Main treatment strategies

All patients received antiviral treatment. The most common antiviral therapy was Lopinavir / Ritonavir alone (70 [29.3%]), Interferon (35 [14.7%]) and their combination (97 [40.8%]). Compared to mild or general patients, less severe or critical patients received Lopinavir / Ritonavir (6 [12.5%] vs 64 [33.7%]; *P*=0.004) and Interferon plus Lopinavir / Ritonavir (5 [10.4%] vs 92 [48.4%]; *P*<0.001). Some patients received Arbidol (16 [6.7%]), Xuebijing (TCM) (17 [7.1%]) and Chloroquine phosphate (3 [1.3%]). More severe or critical patients received Xuebijing (7 [14.6%] vs 10 [5.3%]; *P*=0.03, Table 3). As for the immunosuppressive therapy, 100% severe and critical patients received Glucocorticoid and Gamma-globulin treatment. 28 (14.7%) and 27 (14.2%) mild or general patients received Glucocorticoid and Gamma-globulin treatment, respectively. In addition, all severe and critical patients received pure oxygen inhalation. Four patients received mechanic ventilation support and one patient was treated with ECMO. Two of these four patients died, including the one with ECMO (Table 3).

**Table 3:** Treatment of COVID-19 patients.

	**Total (n=238)**	**Clinical classification**		*P value
		**Mild / General (n=190)**	**Severe / Critical (n=48)**	
Complications, No. (%)	15 (6.3)	3 (1.6)	12 (25.0)	<.001
Antiviral therapy, No. (%)				
Lopinavir/Ritonavir alone	70 (29.3)	64 (33.7)	6 (12.5)	0.004
Lopinavir/Ritonavir + Interferon (analogues)	97 (40.8)	92 (48.4)	5 (10.4)	<.001
Interferon (analogues)	35 (14.7)	32 (16.8)	3 (6.25)	0.06
Arbidol alone	16 (6.7)	11 (5.8)	5 (10.4)	0.33
Arbidol + Interferon (analogues)	11 (4.6)	11 (5.8)	0 (0)	0.13
Xuebijing (TCM)	17 (7.1)	10 (5.3)	7 (14.6)	0.03
Chloroquine phosphate	3 (1.3)	3 (1.6)	0 (0)	>.99
Immunosuppressive therapy, No. (%)				
Glucocorticoid therapy	76 (31.9)	28 (14.7)	48 (100)	<.001
Gamma-globulin therapy	75 (31.5)	27 (14.2)	48 (100)	<.001
Respiratory support, No. (%)				
Oxygen inhalation	48 (20.2)	0 (0)	48 (100)	<.001
Mechanic ventilation	4 (1.7)	0 (0)	4 (8.3)	0.002
ECMO	1 (0.4)	0 (0)	1 (2.1)	0.20

Abbreviations: TCM, traditional Chinese medicine; ECMO, extracorporeal membrane lung oxygenation.

* *P* values indicate differences between mild / general type and severe / critical patients. *P* < .05 was considered statistically significant.

### Treatment outcomes

As of February 27, 2020, 161 patients (67.6%) had been discharged, and 2 patients (0.8%) died.

After admission, 31 mild or general type patients converted to the severe type. Among all these 48 severe or critical patients, 39 (81.3%) severe or critical patients converted to mild or general type after treatment,, at a median of 9 days (IQR, 6-12) after admission. 8 (16.7%) severe patients progressed to critical at a median of 7.5 days (IQR, 2.5-10) after admission, and six of them became better after intensive care and treatment. Two critical patients died. One 64-year Wuhan male died at 16 days after admission, 23 days after he left from Wuhan; one 58-year Changsha male died at 25 days after admission, 34 days after he left from Wuhan.

**Figure 1:**
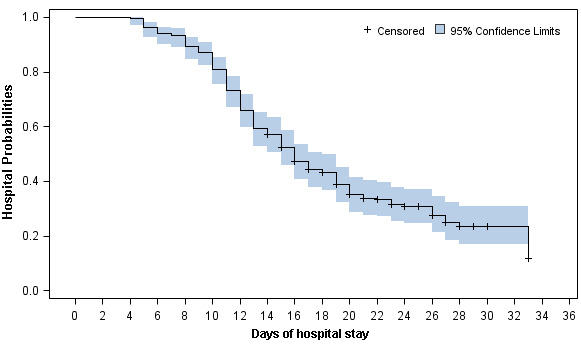
**Kaplan-Meier curve of the hospital probabilities (still in hospitalization without discharge) for all 236 alive hospitalized COVID-19 patients.**

**Figure 2:**
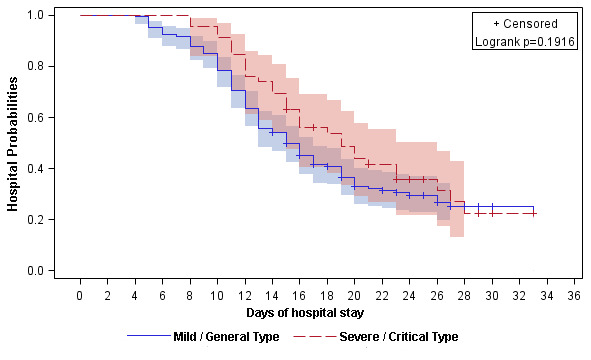
**Kaplan-Meier curve of the hospital probabilities (still in hospitalization without discharge) for alive hospitalized COVID-19 patients stratified by clinical classifications (mild / general and severe / critical).**

The discharge rate was almost the same between mild / general patients and severe / critical patients (130 [68.4%] vs 31 [64.6%]; *P*=0.49). Among those discharged, the duration from admission to discharge was 13 days (IQR, 10-16). Compared to patients with mild or general type, discharged patients who presented with severe or critical type had longer median length of hospital stay (LOH) (12 [IQR, 10-16] days vs 15 [IQR, 12-20]; *P*=0.006) (Table 4). Apart from two dead patients, the Kaplan-Merrier (KM) curve showed the 10-, 14-, 20- and 28-day discharge rate was 19.1%, 42.8%, 65.0% and 76.4%, respectively (Figure 1 and Supplementary Table 1). The 10-, 14-, 20- and 28-day discharge rate for mild / general patients and severe / critical patients were 21.6%, 45.8%, 67.1%, 75.0% and 8.7%, 30.4%, 56.1%, 77.6%, respectively (Logrank *P*=0.19, Figure 2 and Supplementary Table 2).

**Table 4:** Treatment outcome as for February 28, 2020.

	**Total (n=238)**	**Clinical classification**		*P value
		**Mild / General (n=190)**	**Severe / Critical (n=48)**	
Outcome, No. (%)				
Discharge	169 (71)	130 (68.4)	31 (64.6)	0.49
Change to mild/general type	39 (16.4)	0 (0)	39 (81.3)	<.001
Severe change to critical type	8 (3.4)	0 (0)	8 (16.7)	<.001
Death	2 (0.8)	0 (0)	2 (4.2)	0.04
Duration from admission to, median (IQR), d			
Discharge (LOH)	13 (10, 16)	12 (10, 16)	15 (12, 20)	0.006
Change to mild/general type	9 (6, 12)	-	9 (6, 12)	-
Change to critical type	7.5 (2.5, 10)	-	7.5 (2.5, 10)	-
Death, d			20.5 (16, 25)	

Abbreviations: LOH, length of hospitalization.

* *P* values indicate differences between mild / general type and severe / critical patients. *P* < .05 was considered statistically significant.

### The effect of different characteristics on the outcome (discharge)

As for the discharge, the discharge rate is almost the same between severe / critical type and mild / general type. But LOH was significantly longer in severe / critical type. When the discharge is served as an outcome, we performed COX regression analyses to evaluate the effect of antiviral drugs on discharge. Table 5 listed the analysis results of antiviral treatment. Hazard ratio (HR) values indicated the ratio of hazards of discharge among the patients with diverse antiviral therapy compared to the hazards of discharge among the patients with reference Lopinavir/Ritonavir alone treatment. Here, the higher HR is, the more likely to discharge from hospital compared to Lopinavir/ Ritonavir alone treatment. So, Arbitol plus Interferon treatment is significant beneficial for discharge than Lopinavir/Ritonavir alone treatment in total patients (HR (95% CI) = 2.50 (1.07, 5.83), P=0.03). In mild / general patients, Arbitol alone treatment also showed significant beneficial for discharge than Lopinavir/Ritonavir alone treatment (HR (95% CI) = 2.13 (1.08, 4.20), P=0.03). For severe/critical patients, Xuebijing is more beneficial for discharge (HR (95% CI) = 40.99 (2.50, 670.88), P=0.01).

**Table 5:** The impact of different drug management on the prognosis of mild/general and severe/critical patients.

**Candidate variables**	**Total (n=238)**			**Subgroups**				
				**Mild / General (n=190)**		**Severe / Critical (n=48)**	
	*HR (95% CI)	***P* value**	**HR (95% CI)**		***P* value**		**HR (95% CI)**	***P* value**
Antiviral therapy,								
Lopinavir/Ritonavir alone	Ref			Ref			Ref	
Lopinavir/Ritonavir + Interferon (analogues)	1.31 (0.89, 1.93)	0.17		1.29 (0.83, 2.00)	0.25		0.55 (0.07, 4.12)	0.56
Interferon (analogues)	0.95 (0.55, 1.64)	0.85		0.82 (0.45, 1.50)	0.52		3.35 (0.72, 15.65)	0.12
Arbidol alone	1.44 (0.75, 2.75)	0.27		2.13 (1.08, 4.20)	0.03		4.18 (0.51, 34.28)	0.18
Arbidol + Interferon (analogues)	2.50 (1.07, 5.83)	0.03		2.29 (0.89, 5.84)	0.08		-	-
Xuebijing (TCM)	1.51 (0.47, 4.83)	0.49		0.99 (0.24, 4.11)	0.99		40.99 (2.50, 670.88)	0.01
Chloroquine phosphate	0.42 (0.06, 3.07)	0.40		0.40 (0.06, 2.92)	0.37		-	-

Abbreviations: TCM, traditional Chinese medicine, HR, hazard ratio; CI, confidence interval.

*HR values indicated the ratio of hazards of discharge among the patients with diverse antiviral therapy compared to the hazards of discharge among the patients with reference Lopinavir/Ritonavir alone treatment.

In addition, the effects of other clinical characteristics (such as comorbidities and laboratory indexes) on discharge were summarized in Table 6. Most comorbidies seemed to be protective against discharge, but their effects were not significant. Gynecological disease was beneficial for discharge (HR=2.27, P=0.05), but such an effect was unreliable due to minimal sample size (only 7 patients had gynecological disease, Table 1). As for the laboratory indexes, none of them significantly affect the discharge (Table 6).

**Table 6:** The impact of characteristics on prognosis (discharge).

**Candidate variables**	*HR (95% CI)	***P* value**
Age	1.00 (0.99, 1.00)	0.32
Gender		
Female	Ref	
Male	1.14 (0.84, 1.56)	0.41
Type		
Mild / general	Ref	
Severe / critical	0.78 (0.53, 1.15)	0.21
Comorbidities		
Hypertension	0.94 (0.61, 1.44)	0.78
Chronic hepatobiliary disease	0.94 (0.48, 1.84)	0.86
Diabetes	0.96 (0.49, 1.87)	0.89
Heart disease (HD) (8 Coronary HD)	0.90 (0.42, 1.92)	0.78
Pulmonary diseases (1 COPD)	0.83 (0.34, 2.03)	0.69
Cerebrovascular disease	2.10 (0.98, 4.49)	0.06
Bone disease	0.89 (0.39, 2.01)	0.77
Gastric disease	0.63 (0.23, 1.72)	0.36
Gynecological (breast) disease (1 pregnancy)	2.27 (1.00, 5.15)	0.05
Other	1.12 (0.52, 2.38)	0.78
^#^Laboratory Indexes		
White blood cell count	1.09 (0.94, 1.27)	0.24
Neutrophil count	1.04 (0.89, 1.22)	0.59
Lymphocyte count	1.07 (0.95, 1.22)	0.27
Eosinophil count	1.03 (0.91, 1.17)	0.60
Platelet count	1.07 (0.91, 1.25)	0.44
Hemoglobin	0.92 (0.79, 1.06)	0.24

Abbreviations: HR, hazard ratio; CI, confidence interval.

*HR values indicated the ratio of hazards of discharge among the patients with diverse antiviral therapy compared to the hazards of discharge among the patients with reference Lopinavir/Ritonavir alone treatment.

For ^#^Laboratory Indexes, HR (95% CI) was calculated by using the hazards of discharge at 1 standard deviation (SD) increase of the laboratory indexes compared to the hazards of discharge at baseline laboratory indexes.

## DISCUSSION

In this study, we summarized the clinical characteristics and treatment outcome of 236 COVID-19 patients who were diagnosed before February 14 in Changsha city. About 20% patients were severe and critical type. Two patients died due to the severe disease. The 10-, 14-, 20- and 28-day discharge rate was 19.1%, 42.8%, 65.0% and 76.4%, respectively. The 10-, 14-, 20- and 28-day discharge rate for mild / general patients and severe / critical patients were 21.6%, 45.8%, 67.1%, 75.0% and 8.7%, 30.4%, 56.1%, 77.6%, respectively.

Compared to patients with mild or general type, discharged patients who presented with severe or critical type had longer median length of hospital stay (LOH) (12 [IQR, 10-16] days vs 15 [IQR, 12-20]; *P*=0.006). In addition, severe / critical patients were older, more likely to visit Wuhan, get in touch with Wuhan people. Mild / general patients were more likely to be infected by patients and family members. Severe/critical patients were more likely to have comorbidities, such as hypertension, heart disease and chronic kidney disease. Severe/critical patients were more likely to have symptoms, such as fever, fatigue, dyspnea, etc. Moreover, severe / critical patients had lower lymphocyte and platelet counts. TCM Xuebijing were used more frequently in severe / critical patients and this drug showed significant benefit in severe / critical patients.

The coronavirus disease-19 (COVID-19) spreads rapidly and has obvious family aggregation [7]. Currently, there are no specific antiviral drugs to kill the virus (treatment guideline). Comprehensive management and active symptomatic treatment are the main treatment strategies. As COVID-19 is a respiratory infectious disease with strong transmission, we need high personal protection requirements for doctors and nurses. Comprehensive strategies have played important roles in decreasing the mortality rate and preventing the infection of medical workers. Here, we summarized three main strategies, including the treatment strategy, infection control strategy, and safeguard strategy.

### The first one is the treatment strategy

1.1 For mild patients, doctor in charge assessed patient’s condition daily according to the patient’s symptoms, vital signs and oxygen saturation. Patients who had no fever at 3-5 days after hospitalization received coronavirus nucleic acid tests and CT scan for doctors to detect disease changes. As shown in Table 1, 160 (67.2%) cases reported having fever before hospitalization, but the majority of patients had no fever after admission. As shown in Table 2, only 58 (24.3%) patient had fever and most of them (42 [72.4%]) were low-fever (37.3-38.0 °C).

Among 94 patients who had no fever after admission, 16 patients were found obvious glass-like changes in bilateral lungs by CT scan at three days later post-admission. That was a sign for disease exacerbation from mild to severe. At that moment, the addition of pure oxygen inhalation, low-dose glucocorticoid and short-term gamma-globulin therapy was necessary. By above treatment, 15 patients turned back to mild, 10 of whom turned to mild in 2-6 days; the rest 5 patients turned to mild in 15-20 days. However, one patient became worsen to critical. With active treatment, he had turned back to severe type and still stayed in hospital. Due to the CT finding at early stage, we could detect the changes in lung and perform active treatment as early as possible, which effectively shortened the duration of patients in severe condition. For patients who had unilateral glass-grinding change by CT scan at 3 days post-admission, oxygen inhalation and active symptomatic treatment were given to timely prevent the transition of mild disease to severe disease. For patients who were negative in coronavirus nucleic acid test after antiviral therapy for long time, we collected their blood serum and stem cells at the recovery stage. As for February 27, the accumulative number of discharge patients was 161. The cure rate was 67.6%. The average length of hospital stay was 12.5 days. Among cities which had accumulative confirmed COVID-19 patients of more than 200 cases, Changsha’s discharge rate ranks 5^th^.

1.2 For severe and critical patients, their condition changed rapidly and they had more basic diseases. Therefore, the treatment was more difficult than mild patients. In this case, we centralized our medical recourses, experts, drugs and patients. All severe and critical patients were admitted in two ICU wards, and all medical professionals experienced in intensive medicine were concentrated in these two wards. We adopted the ‘one-person-one-team’ strategy to secure every severe or critical patient to have his/her own team of doctor and nurse. Every day, the team leader must report all the patient’ situation to the senior doctors, including the vital signs, blood gas analysis results, changes in biochemical indexes and clinical symptoms, and airway management, etc. For patient who poorly responded to treatment, the onsite senior doctors need to provide alternative effective options as soon as possible. For patients who had basic diseases or complications, senior specialists would give their corresponding treatment advice. For patients who might became severe or critical according to image and laboratory alert, a group of senior experts would be invited through remote consultation system for next treatment regimen. Our senior experts were from three Xiangya affiliated hospitals, Hunan University of traditional Chinese Medicine, and Hunan Institute of traditional Chinese Medicine, etc.

1.3 As for the traditional Chinese medicine (TCM), more than 90% cases received TCM treatment. TCM experts differentiated symptoms and exerted treatment for patients daily. We found that COVID-19 in Changsha area had some regularity in TCM pathogenesis, which belonged to the ‘warm heat’ type of epidemic disease. On the whole, it could be treated according to TCM ‘warm’ epidemic disease; but there was also a great degree of variability, especially for patients with basic disease, old patients, and severe / critical patients. The TCM syndrome type of COVID-19 was closely related to its basic constitution, which accorded with the theory that "the external evil is moving, the recipient is hard to know; patient being aware once having symptoms, then disease could be distinguished ". From the real-world observation, among COVID-19 patient with mild and general type, TCM syndrome types ‘warm evil attacked defense system’, ‘warm dryness injured saliva’, and ‘little sun stagnated heat’ were common, and each syndrome type was also always coupled with ‘dampness evil (or turbid poison)’. For severe patients, common TCM syndrome types were ‘gas-water deficiency’, ‘evil heat blocked lung’, and ‘heat phlegm accumulated in lung’. For critical patients, the syndrome type and performance were ‘extreme gas-water deficiency’, ‘internal closed with external collapsed’.

1.4 Nutrition support and early rehabilitation training were important for critical patients. Nasal jejuna nutrition-feeding tube could effectively reduce the risk of reflux and aspiration, and improve the tolerance of patients to enteral nutrition (EN). Early rehabilitation training has pretty high potency ratio, because it could reduce the mortality of severe patients and shorten the length of hospital stay and length of ventilator use. But critical patients were not able to rehabilitate actively, so medical staff need to intervene proactively. COVID-19 patients, especially the severe patients, can achieve good outcome by early lung rehabilitation. Early lung rehabilitation (ELR) is good for sputum drainage and functional exercise of diaphragm; ELR can prevent thrombosis, re-infection and other complications; it is also conducive to control pneumonia, prevent ventilator-associated pneumonia, reduce the risk of deep venous thrombosis, and improve mental health and life quality.

1.5 Psychological care is particularly important in severe patients and those with positive viral nucleic acid for long time. On the one hand, they are concerned that the disease cannot be effectively controlled; on the other hand, they are concerned about the medication-caused side effects and sequelae after discharge. Most of these patients are highly educated and are more stressed than the average, so psychological care is particularly important. The measures we take are to send them daily greeting messages to relieve their psychological stress, eliminate their fear and improve their compliance with medication.

2. The second strategy is the infection control strategy. We have taken the following measures to prevent infection of medical workers:

2.1. We strictly set up individual ‘three zones’ (contaminated zone, potential contaminated zone, and clean zone) and ‘two channels’ (medical personnel passage and patient passage). All items must be passed through the transfer window, which is sterilized by ultraviolet light.

2.2 We setup dressing mirror for workers to check protective cloth by themselves. We also have full-time supervisor to double check the guard suite. In the clothes taking-off room, the clothes taking-off process is printed out on the wall, and surveillance cameras monitor the taking-off process. Medical workers who are going to leave the contaminated zone should be taken out in pairs for mutual supervision. Medical workers must bathe and change clothes before leaving the ward. Then, they stay in a designated hotel to rest.

2.3 Sufficient rest time and psychological care are necessary for medical workers. All medical staffs work 4 to 6 hours per shift. Anyone who has physical discomfort must leave the isolation ward and take a rest at the designated hotel. There are ‘three not-allowed’: 1) sick personnel is not allowed to work in isolation ward, 2) fatigue ones are not allowed to work, 3) workers without correct dressing are not allowed to work. Professional psychological counselors carry on psychological counseling for medical workers in designated hospital to relieve their panic mental.

2.4 To ensure the personal protection of medical workers who take care of patients during transporting, all patient-transporting vehicles are negative pressure ambulances.

**Figure 3:**
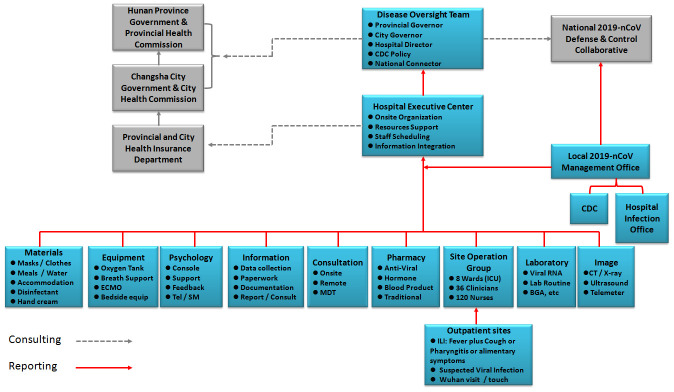
**Organization structure of COVID-19 defense and control system.**

3. The third one is the material support strategy. As shown in Figure 3, we have a strong material support system, including the net-work support system. To ensure the treating capacity of 300 patients, we renovated three floors in two buildings and opened eight nursing unit within 6 days immediately after Spring Festival. All the patients in Changsha area are timely admitted and treated in our hospital. All related departments, including the Finance Bureau, Health Commission, Residential and Construction Bureau, Environmental Protection Department, Gas Provider, Bureau of Governmental Affairs, CDC, etc, have made their best to contribute and ensure the hospitalization of all infected patients and zero-hospital infection of medical staff. In short period after COVID-19 outbreak, we selected and trained 600 medical staffs from the public hospitals throughout the city. We arranged them to work in isolation ward by stages and in groups. We also purchased all the protective materials and medical equipment in emergency, including ECMO, broncho-fiberscope, mobile CT, etc. We requisitioned three hotels for medical staffs to take a rest, and two hotels to centralize the discharged patients for isolation and clinical observation. To secure enough rest for medical staffs, nine buses are responsible for the daily shifting, according to the medical staffs’ working time schedule. Bureau of Environmental Protection and CDC are in charge of properly dealing with the medical sewage and trash. The gas, electricity, water and telecom providers ensure the sufficient resource supply. Governmental Affair Bureau guarantees the daily diet and nutrition of all medical staffs and patients. During the entire medical treatment process, it is precisely because of such a safeguard support system, we have no worries, and we spend all our time and energy on how to rescue, treat and care patients, how to do a good job for personal protection and prevent infection for workers. We make our best to control mild patients not to become severe, severe patients not to become critical, and critical patients not to die.

As of February 27, 2020, two patients have died. One 64-year old male patient died on February 15, 2020. This patient had hypertension, COPD and smoking history. Multilobular infiltration, lymphopenia and bacterial co-infection occurred during disease progression. This patient had all six indexed in the MuLBSTA score [7], which is effective in predicting mortality in viral pneumonia. Another 58-year old male patient died on February 21, 2020. This patient left Wuhan on January 14, 2020, and was diagnosed and admitted on January 23, 2020. At admission, this patient had lung infiltration by CT scan, belonging to general type. On January 31, 2020, the disease progressed rapidly and critically. After 16-days of treatment, with invasive ventilation CRRT and even ECMO, this patient died on February 17, 2020.

## MATERIALS AND METHODS

### Study design and participants

This study was approved by the institutional ethics board of the First Hospital of Changsha city (No. KL-2020002). All consecutive patients with confirmed COVID-19, who were admitted to the north hospital of First Hospital of Changsha city from January 22 to February 14, 2020, were enrolled. Signed consent was obtained from patients. The First Hospital of Changsha city is the teaching hospital of Central South University and Nanhua University. Its north hospital was established in response to SARS in 2003. Currently, the north hospital of the First hospital of Changsha city is the only designated hospital responsible for the treatments for all COVID-19 patients throughout Changsha city. All COVID-19 patients were diagnosed according to WHO interim guidance [8]. All COVID-19 patients admitted in the north hospital were treated in accordance with the national COVID-19 treatment guidance (Trial Edition 2). Consistent with the published Wuhan study [4], the treatment outcomes were discharge, mortality, and length of hospital stay (LOH). All patients were followed up until February 27, 2020.

### Diagnostic criteria

Suspected cases were diagnosed according to the clinical manifestations combined with the following epidemiological histories: 1) travel history or residence history in the community of Wuhan and its surrounding areas within 14 days prior to onset of the disease; 2) contact history with COVID-19 (RT-PCR 2019-nCoV positive) patient within 14 days before onset; 3) contact with people who had fever or respiratory symptoms and migrated from Wuhan and the surrounding areas or from the community where COVID-19 cases have been reported, and contact with people who had fever or respiratory symptoms within 14 days prior to onset of the disease; (4) cases with cluster disease. The clinical manifestations included: 1) fever and/or respiratory symptoms; 2) imaging features of coronavirus pneumonia [9]; 3) the total number of white blood cells (WBC) was abnormal or decreased, or the count of lymphocytes was reduced. Patients with any one of the epidemiological history combined with any 2 of the clinical manifestations, or patients without a clear epidemiological history but having all three clinical manifestations, were diagnosed as the suspected patients.

Confirmed cases were suspected cases with one of the following pieces of etiological evidence [10, 11]: 1) detection of 2019-nCoV positive by real-time fluorescence RT-PCR in respiratory or blood samples; 2) Sequencing of the virus genes in respiratory or blood samples, highly homologous to the known 2019-nCoV.

### Discharge criterion and follow-up procedure

In clinical practice, the discharge time for all patients was 1-2 days after 2019-nCoV nucleic acid test changed to negative. As for the image criterions, 1) for severe patients without underlying disease, the discharge time was after CT scan showed that the pneumonia lesion was almost absorbed; 2) for general patients, the lung lesion should be totally absorbed before discharge. All discharged patients should be followed up with every 5 days for 30 days after discharge. All discharged patients should be self-isolated at home for the first 14 days after discharge. A psychologist was responsible for contacting the discharged patients to relieve their stress.

### Data collection

For all patients, the basic demographics, medical history and epidemiological information, including age, gender, occupation, disease history, living place (province, city, district, etc), Wuhan visit history, disease exposure history, family exposure history, were collected at admission. We recorded the pre-admission influenza-like illness (ILI) [12] symptoms (fever, cough, pharyngitis, diarrhea, etc) at admission. After admission, we recorded all the examination and treatment information, including the physical examination findings, laboratory and image findings, complications, symptoms, pharmaceutical applications, respiration support, etc. All data were collected by the medical records office. All patients’ medical records were analyzed by a group of professional statistical analysts from Central South University, Emory University and ICF.

### Clinical classifications

According to the clinical features, COVID-19 patients were categorized into mild, general, severe and critical type [13, 14]. Mild patients only showed slight fever and mild fatigue without pneumonia CT change [15, 16]. The general patient had fever, respiratory symptoms, and a CT scan detecting featured pneumonia change [14]. Severe patients often had dyspnea and/or hypoxemia within one week after the onset of the disease, and severe patients quickly progressed to acute respiratory distress syndrome (ARDS), septic shock, metabolic acidosis and coagulation dysfunction [17]. The severe disease was identified once any of the following criteria was met: 1) respiration distress (respiration rate > 30 / min); 2) at rest, the oxygen saturation < 93%; 3) the partial pressure of arterial blood oxygen (PaO_2_) / Fraction of inspiration O_2_ (FiO_2_) < 300mmHg. The critical disease was identified once any of the following criteria was met: 1) Respiratory failure and patient needed the mechanical ventilation; 2) the appearance of shock; 3) ICU monitoring was required for the combination of other organ failure. It is worth noting that the severe and critical patients in the course of the disease could be with slight fever, or even no obvious fever ^10^.

### Statistical analysis

Continuous variables were summarized in terms of the median (interquartile range). Categorical variables were described via frequencies and percentages. Independent group t-tests were used to compare continuous variables where data were approximately normally distributed; otherwise, the Mann-Whitney test was used. Mixed linear models were used for repeated records. Chi-square tests were used to compare the proportions for categorical variables; Fisher’s exact test was adopted for limited subgroups. Cox regression analysis was used to evaluate the univariate and multivariate risk of candidate gene mutations in progression. The Kaplan-Meier method was used to estimate the survival distributions against progression, and the log-rank test was used to assess differences in PSF experience among subgroups. All tests of hypotheses were two-tailed and conducted at a significance level of 0.05. Statistical analyses were conducted using SAS 9.4.

## Supplementary Material

Supplementary Tables
