# Correction for: Treatment strategies of hospitalized patients with coronavirus disease-19

**DOI:** 10.18632/aging.203034

**Published:** 2021-04-29

**Authors:** Yaxiong Huang, Chunlin Cai, Jinglei Zang, Jun Xie, Dan Xu, Fang Zheng, Tao Zhan, Kang Huang, Yikai Wang, Xiao Wang, Zhe-Yu Hu, Yapeng Deng, Yuanlin Xie

**Affiliations:** 1 The First Hospital of Changsha City, Changsha, Hunan 410005, China; 2 Changsha Health Vocational College, Changsha, Hunan 410100, China; 3 Emory University Rollins School of Public Heath, Atlanta, GA 30322, USA; 4 ICF, 3 Corporate Square NE, Atlanta, GA 30329, USA; 5 Hunan Cancer Hospital, The Affiliated Cancer Hospital of Xiangya School of Medicine, Central South University, Changsha, Hunan 410013, China; 6 The Forth Hospital of Changsha City, Changsha, Hunan 410013, China

**Keywords:** correction

Original article: Aging. 2020; 12:11224–11237.  . https://doi.org/10.18632/aging.103370

**This article has been corrected:** The authors neglected to include the funding of Changsha City Technology Program for Zhe-Yu Hu. The authors state that this correction does not change the scientific conclusions of the article. The updated Funding information is provided below.

