# Supplementary material for: Treatment strategies of hospitalized patients with coronavirus disease-19
Source: Aging (Albany NY). 2020 Jun 17;12(12):11224–37. doi: 10.18632/aging.103370 (PMC7343487; doi:10.18632/aging.103370)
Supplement: Supplementary Tables [file aging-12-103370-s001..pdf]

## SUPPLEMENTARY TABLES

**Supplementary Table 1. Discharge rate and hospitalization rate information for 236 alive NCIP patients, as of February 27, 2020.**

| Timelist (days) | Hospital stay time | Hospitalization probability | Discharge probability | Discharged | Number Left |
|---|---|---|---|---|---|
| 0 | 0 | 1 | 0 | 0 | 236 |
| 2 | 0 | 1 | 0 | 0 | 236 |
| 4 | 4 | 0.9958 | 0.00424 | 1 | 235 |
| 6 | 6 | 0.9407 | 0.0593 | 14 | 222 |
| 8 | 8 | 0.8941 | 0.1059 | 25 | 211 |
| 10 | 10 | 0.8093 | 0.1907 | 45 | 191 |
| 12 | 12 | 0.6610 | 0.3390 | 80 | 156 |
| 14 | 14 | 0.5720 | 0.4280 | 101 | 129 |
| 16 | 16 | 0.4719 | 0.5281 | 123 | 99 |
| 18 | 18 | 0.4334 | 0.5666 | 131 | 86 |
| 20 | 20 | 0.3503 | 0.6497 | 147 | 61 |
| 22 | 22 | 0.3329 | 0.6671 | 150 | 54 |
| 24 | 24 | 0.3073 | 0.6927 | 154 | 37 |
| 26 | 26 | 0.2755 | 0.7245 | 157 | 22 |
| 28 | 28 | 0.2365 | 0.7635 | 160 | 15 |
| 30 | 28 | 0.2365 | 0.7635 | 160 | 2 |
| 32 | 28 | 0.2365 | 0.7635 | 160 | 2 |
| 34 | 33 | | | 161 | 0 |

**Supplementary Table 2. Discharge rate and hospitalization rate information for 190 mild / general NCIP patients and 46 alive severe / critical NCIP patients, as of February 27, 2020.**

| Timezlist (days) | Mild / General Type | | | | | Severe / Critical Type | | | | |
|---|---|---|---|---|---|---|---|---|---|---|
| | Hospital stay time | Hospitalization probability | Discharge probability | Discharged | Number Left | Hospital stay time | Hospitalization probability | Discharge probability | Discharged | Number Left |
| 0 | 0 | 1 | 0 | 0 | 190 | 0 | 1 | 0 | 0 | 46 |
| 2 | 0 | 1 | 0 | 0 | 190 | 0 | 1 | 0 | 0 | 46 |
| 4 | 4 | 0.9947 | 0.00525 | 1 | 189 | 0 | 1 | 0 | 0 | 46 |
| 6 | 6 | 0.9263 | 0.0737 | 14 | 176 | 0 | 1 | 0 | 0 | 46 |
| 8 | 8 | 0.8789 | 0.1211 | 23 | 167 | 8 | 0.9565 | 0.0435 | 2 | 44 |
| 10 | 10 | 0.7842 | 0.2158 | 41 | 149 | 10 | 0.9130 | 0.0870 | 4 | 42 |
| 12 | 12 | 0.6368 | 0.3632 | 69 | 121 | 12 | 0.7609 | 0.2391 | 11 | 35 |
| 14 | 14 | 0.5421 | 0.4579 | 87 | 97 | 14 | 0.6957 | 0.3043 | 14 | 32 |
| 16 | 16 | 0.4506 | 0.5494 | 103 | 75 | 16 | 0.5604 | 0.4396 | 20 | 24 |
| 18 | 18 | 0.4083 | 0.5917 | 110 | 64 | 18 | 0.5360 | 0.4640 | 21 | 22 |
| 20 | 20 | 0.3287 | 0.6713 | 122 | 43 | 20 | 0.4386 | 0.5614 | 25 | 18 |
| 22 | 22 | 0.3134 | 0.6866 | 124 | 39 | 21 | 0.4142 | 0.5858 | 26 | 15 |
| 24 | 24 | 0.2958 | 0.7042 | 126 | 26 | 23 | 0.3590 | 0.6410 | 28 | 11 |
| 26 | 26 | 0.2677 | 0.7323 | 128 | 15 | 26 | 0.3141 | 0.6859 | 29 | 7 |
| 28 | 27 | 0.2498 | 0.7502 | 129 | 10 | 28 | 0.2244 | 0.7756 | 31 | 5 |
| 30 | 27 | 0.2498 | 0.7502 | 129 | 1 | 28 | 0.2244 | 0.7756 | 31 | 1 |
| 32 | 27 | 0.2498 | 0.7502 | 129 | 1 | 28 | 0.2244 | 0.7756 | 31 | 1 |
| 34 | 33 | 0 | 1.0000 | 130 | 0 | 28 | | | 31 | 0 |
